# A gut-oral microbiome–driven axis controls oropharyngeal candidiasis through retinoic acid

**DOI:** 10.1172/jci.insight.160348

**Published:** 2022-09-22

**Authors:** Felix E.Y. Aggor, Martinna Bertolini, Chunsheng Zhou, Tiffany C. Taylor, Darryl A. Abbott, Javonn Musgrove, Vincent M. Bruno, Timothy W. Hand, Sarah L. Gaffen

**Affiliations:** 1 Division of Rheumatology & Clinical Immunology, Department of Medicine, and; 2 Department of Periodontics and Preventive Dentistry, School of Dental Medicine, University of Pittsburgh, Pittsburgh, Pennsylvania, USA.; 3 Richard King Mellon Foundation Institute for Pediatric Research, Department of Pediatrics, UPMC Children’s Hospital of Pittsburgh, University of Pittsburgh, Pittsburgh, Pennsylvania, USA.; 4 Institute for Genome Sciences, University of Maryland School of Medicine, Baltimore, Maryland, USA.

**Keywords:** Immunology, Cytokines, Fungal infections

## Abstract

A side effect of antibiotics is outgrowth of the opportunistic fungus *Candida albicans* in the oropharynx (oropharyngeal candidiasis, OPC). IL-17 signaling is vital for immunity to OPC, but how the microbiome impacts antifungal immunity is not well understood. Mice in standard specific pathogen–free (SPF) conditions are resistant to OPC, whereas we show that germ-free (GF) or antibiotic-treated mice are susceptible. Oral type 17 cells and IL-17–dependent responses were impaired in antibiotic-treated and GF mice. Susceptibility could be rescued in GF mice by mono-colonization with segmented filamentous bacterium (SFB), an intestine-specific constituent of the microbiota. SFB protection was accompanied by restoration of oral IL-17^+^CD4^+^ T cells and gene signatures characteristic of IL-17 signaling. Additionally, RNA-Seq revealed induction of genes in the retinoic acid (RA) and RA receptor–α (RARα) pathway. Administration of RA rescued immunity to OPC in microbiome-depleted or GF mice, while RAR inhibition caused susceptibility in immunocompetent animals. Surprisingly, immunity to OPC was independent of serum amyloids. Moreover, RAR inhibition did not alter oral type 17 cytokine levels. Thus, mono-colonization with a component of the intestinal microflora confers protection against OPC by type 17 and RA/RARα, which act in parallel to promote antifungal immunity. In principle, manipulation of the microbiome could be harnessed to maintain antifungal immunity.

## Graphical abstract

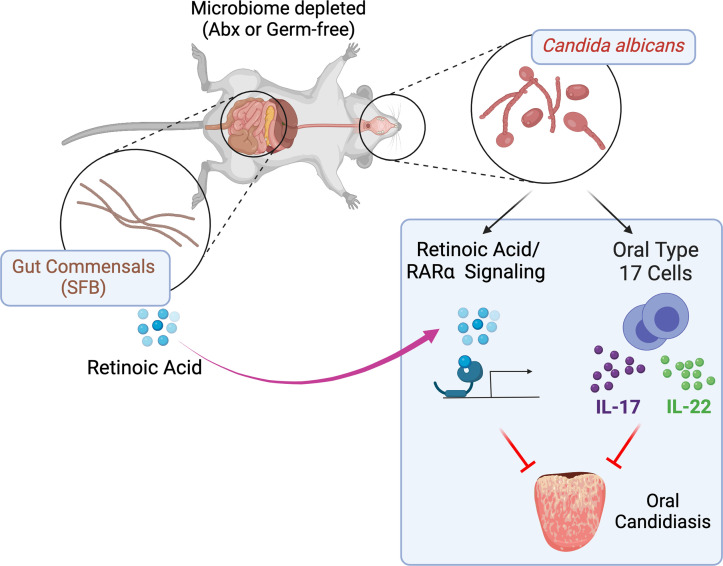

## Introduction

Oropharyngeal candidiasis (OPC, oral thrush) is an opportunistic fungal infection of the oral and esophageal mucosa caused by *Candida albicans*. OPC is seen in HIV/AIDS, treatment with immunosuppressants, and inadequate immune responses in infants and the aged (1, 2). Although not life-threatening, severe OPC can cause considerable morbidity and is linked to nutritional deficits, failure to thrive, and increased incidence of esophageal cancer (3–5). Although infections can often be controlled with antifungal drugs such as azoles, microbial resistance to such drugs is on the rise (6, 7), and alternative strategies to treat fungal infections represent an unmet clinical need.

In both mice and humans, protection against OPC is mediated by signaling in oral epithelial cells through the cytokine IL-17 via its essential downstream adaptor NF-κB activator 1 (Act1) (8–13). Additionally, IL-22 and STAT3 in oral epithelial cells drive mucosal responses to oral candidiasis (8, 14, 15). IL-17 and IL-22 act on distinct layers of the stratified oral epithelium, with IL-17 required largely in the superficial postmitotic epithelial cell layer and IL-22/STAT3 acting on the proliferative basal epithelial layer (15). Mutations in genes encoding the IL-17 receptor or Act1, or genes important for Th17 differentiation such as RAR related orphan receptor C (RORC, encoding the transcription factor RORγt), all predispose to chronic mucocutaneous candidiasis in mice and humans, demonstrating that the mouse is a fairly faithful model of events that promote immunity to this fungus (reviewed in refs. 16, 17). Despite these advances, our understanding of the mechanisms of immunity within the oral cavity remains incomplete, and there are no licensed vaccines to any fungal pathogens (18, 19).

The microbiome exerts widespread influences on control and development of immune responses and has exciting potential for translational interventions (20, 21). However, most studies of mucosal biology are performed in the context of the gastrointestinal tract, and the influence of intestinal microbiota on extraintestinal sites remains an area of active inquiry. In this regard, microbiome-depleted mice infected with *C*. *albicans* show elevated and prolonged colonization in the gut, with profound impacts on systemic immune responses and other physiological events (22–24). With respect to OPC, antibiotics are known to confer disease susceptibility in humans and mouse models (25).

While mucosal immunology of the intestine is intensely studied, our understanding of oral mucosal immunity and of the influence of oral and gut microbiomes in shaping oral immune responses is comparatively rudimentary (26, 27). Intriguingly, in the context of periodontitis, development of gingiva-resident Th17 cells is driven by masticatory tissue damage rather than the microbiome (28). Even so, the oral cavity is home to a rich and unique microbiome, perturbations of which can cause fungal susceptibility (29). There are emerging insights regarding an oral-gut microbiome axis. Oral dysbiosis in periodontitis supplies oral microbial antigens and Th17 cells to the gut. These in turn activate oral pathobiont-reactive Th17 cells that exacerbate gut inflammation (30). It is therefore apparent that type 17 responses in the oral mucosa are compartmentalized and may be affected by both oral and gut microbiota.

An influential component of the murine intestinal microbiome is segmented filamentous bacterium (SFB), an anaerobic, Gram-positive microbe that colonizes the distal ileum (31–34). SFB adheres to intestinal epithelial cells and and potently activates Th17 cells. Despite being restricted to the gut (35), SFB activates many extraintestinal events driven by Th17 cells (36, 37). For example, gnotobiotic mono-colonization models using SFB have demonstrated capacity to exacerbate Th17-driven autoimmune models of arthritis and diabetes (31, 38). Similarly, SFB can confer protection against bacterial and viral infections (39, 40).

Here, we took advantage of a murine model of OPC to understand if this gut-restricted member of the intestinal microbiota can influence oral immunity and whether this axis could be exploited for therapeutic benefit. Unlike mice with a replete microbiome, gnotobiotic (germ-free, GF) mice or mice treated with antibiotics were highly susceptible to OPC. Strikingly, SFB mono-colonization in GF mice was sufficient to restore immune protection. SFB administration rescued expression of IL-17 and IL-22 in oral lymphocytes, accompanied by upregulation of a characteristic type 17 gene signature in the oral mucosa, including β-defensins and neutrophil-recruiting chemokines. SFB mono-colonization also induced gene networks indicative of epithelial repair and keratinization as well as a panel of retinol (vitamin A) metabolism pathway genes. Indeed, administration of retinoic acid (RA) in antibiotic-treated or GF mice reversed susceptibility to OPC, whereas inhibition of RA receptor–α (RARα) provoked susceptibility to disease in immunocompetent SPF mice. Surprisingly, RA administration did not alter type 17 levels, and serum amyloids (SAAs) were dispensable for immunity to OPC, despite their capacity to deliver retinol and promote type 17 immunity (41). Thus, a single member of the intestinal commensal microbiome has the capacity to confer protective immune responses in the oral cavity.

## Results

### Microbiome depletion impairs oral type 17 responses and elevates susceptibility to OPC.

**Figure 1:**
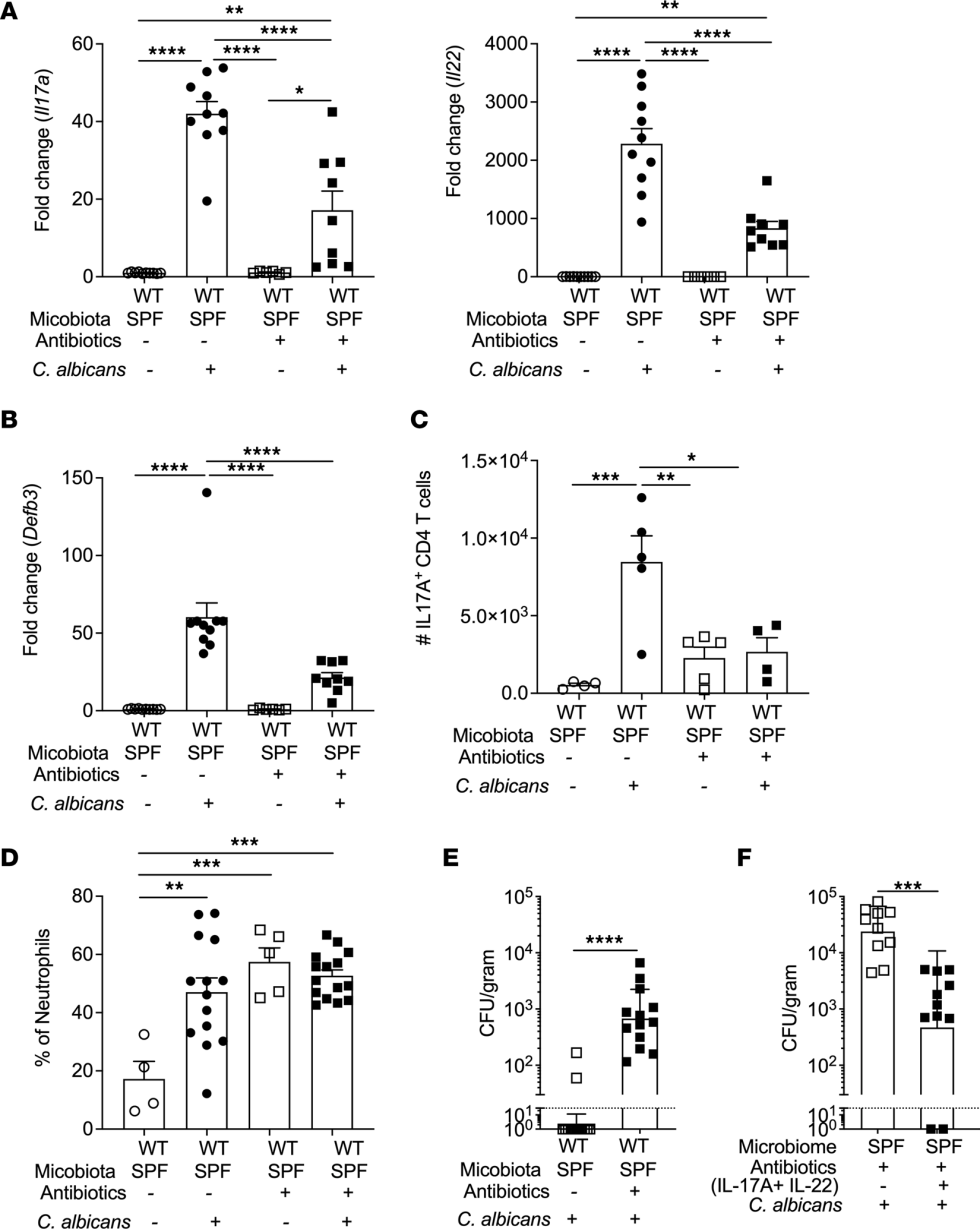
Microbiome depletion impairs type 17 responses in OPC. (**A** and **B**) WT mice in SPF conditions (WT-SPF), ages 6–9 weeks, both sexes, were treated with or without antibiotics in drinking water for 7 days and infected orally by a 75-minute sublingual exposure to *C*. *albicans* or PBS (*n* = 9–12 mice/group). RNA from whole tongue prepared at day 2 p.i. was subjected to quantitative PCR (qPCR) for the indicated genes normalized to *Gapdh*. Graphs show mean ± SEM. Data are pooled from 2 independent experiments. Mice were infected and on day 2 p.i. tongue homogenates were analyzed by flow cytometry to assess (**C**) IL-17A production (CD4^+^IL17A^+^, gated on live CD45^+^ cells) and (**D**) neutrophils (CD11b^+^Ly6G^+^ gated on live CD45^+^ cells). (**E**) Mice were infected as in **A**, and on day 5 p.i. fungal burdens were assessed in tongue homogenates by plating and CFU enumeration. Graphs show geometric mean ± SD. Dashed line indicates limit of detection. Data were pooled from 2 independent experiments. (**F**) Mice treated as in **A** were administered recombinant IL-17A and IL-22 i.p. Fungal burdens were assessed at day 5. Dashed line indicates limit of detection. Data were pooled from 2 independent experiments. Data analyzed by ANOVA with Tukey’s multiple comparisons test (**A**–**D**) or Mann-Whitney *U* test (**E** and **F**). *P < 0.05, ** < 0.01, *** < 0.001, **** < 0.0001.

Considerable evidence supports a role for commensal bacteria in maintaining immunity to *C*. *albicans* in the oral mucosa. Mice do not harbor *C*. *albicans* as a commensal microbe and thus are immunologically naive to this fungus (9). Nonetheless, WT mice raised in standard specific pathogen–free (SPF) housing exhibit rapid, potent resistance to oral *C*. *albicans* infection, clearing fungi within 3 days postinfection (p.i.) and showing no overt disease (42–44). To understand how the microbiome promotes responses to OPC, C57BL/6 mice of both sexes were housed in SPF conditions (WT-SPF) and were given broad-spectrum antibiotics in drinking water (Abx; metronidazole, ampicillin, neomycin, vancomycin). After 7 days, mice were subjected to a 75-minute sublingual infection with *C*. *albicans* strain CAF2-1 (42, 45). After 2 days, tongue mRNA was evaluated for genes characteristic of the Th17/IL-17 pathway known to be important in immunity to OPC. As previously shown, *Il17a* and *Il22* were highly upregulated in the tongue at this time point (15, 46). However, *Il17a* and *Il22* levels were substantially lower in Abx-treated mice (Figure 1A). Consistent with this, expression of the IL-17–dependent antimicrobial peptide β-defensin 3 (BD3, *Defb3*) was also reduced (Figure 1B). Commensurate with these observations, the total number of IL-17–expressing CD4^+^TCRαβ^+^ lymphocytes in the tongue was reduced upon microbiome depletion (Figure 1C). IL-17 promotes neutrophil accumulation during OPC by inducing CXC chemokine expression in oral epithelia (43, 47). Surprisingly, neutrophil recruitment to the tongue was increased upon Abx treatment (Figure 1D), potentially due to the elevated fungal loads in Abx-treated mice. Thus, microbiome depletion caused by Abx impairs development of some but not all type 17–driven antifungal pathways.

To determine the impact of Abx treatment on susceptibility to overt OPC, WT-SPF mice were given Abx, then infected orally with *C*. *albicans*, and fungal burdens in tongue were assessed on day 5 by serial dilution plating (limit of detection: ~30–50 CFU/g tissue) (48). As expected, mice without antibiotics cleared *C*. *albicans* completely, whereas Abx treatment impaired fungal clearance in all mice tested, with fungal loads averaging approximately 10^3^ CFU/g (Figure 1E). To determine if type 17 cytokines were sufficient to rescue the effects of microbiome depletion, recombinant IL-17 and IL-22 were injected i.p. 12 and 24 hours prior to oral *C*. *albicans* infection and fungal loads assessed at day 5. Interestingly, while IL-17 and IL-22 administration significantly reduced fungal burdens in microbiome-depleted mice, very few animals (2 out of 11) cleared *C*. *albicans* (Figure 1F), indicating that in addition to type 17–mediated protection, other factors appear to be needed to allow *C*. *albicans* clearance in the absence of a normal microbiome.

### Mono-colonization with SFB protects against OPC.

**Figure 2:**
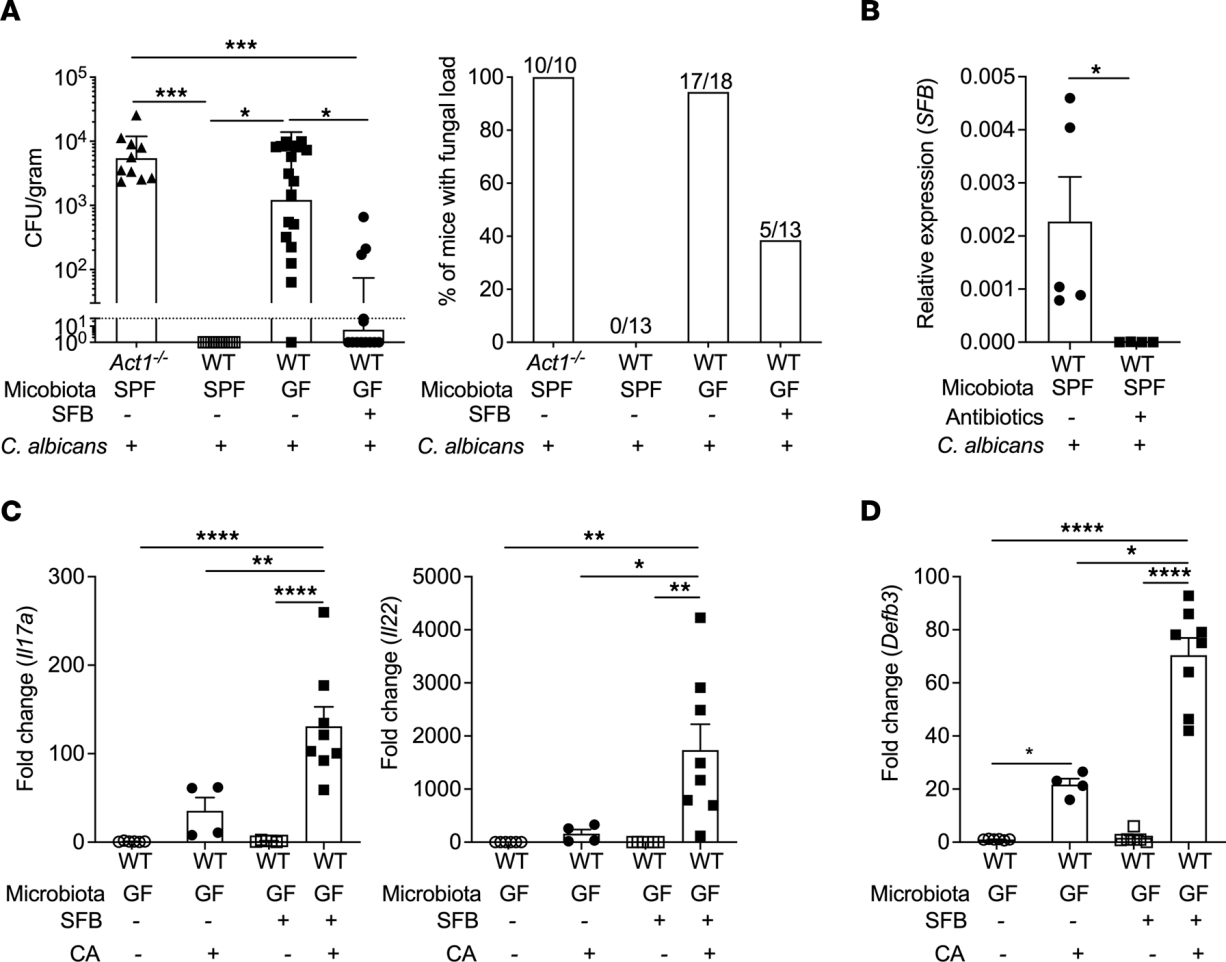
Mono-colonization with SFB induces protective responses to oral candidiasis in GF mice. (**A**) The indicated mice (SPF or GF) were gavaged with SFB or PBS. After 14 days, mice were infected orally with *C*. *albicans* and fungal burdens assessed at day 5 by plating and CFU enumeration. *n* = 4–15 mice/group. *Left*: Geometric mean ± SD and analyzed by ANOVA and Tukey’s multiple comparisons test. Data pooled from 3 experiments. *Right*: Percentage clearance (number of mice with detectable fungal load/total mice). (**B**) WT-SPF mice were treated with antibiotics in drinking water or left untreated for 7 days. Fecal SFB content was analyzed by qPCR and normalized against total fecal bacterial content. Data from 1 experiment. (**C** and **D**) RNA from whole tongue at day 2 p.i. was subjected to qPCR for the indicated genes. Graphs show mean ± SEM and data were analyzed by ANOVA and Tukey’s multiple comparisons test. Data pooled from 2 experiments. *P < 0.05, ** < 0.01, *** < 0.001, **** < 0.0001.

Even broad-spectrum antibiotics do not fully eliminate commensal microbiota, so we next assessed immunity to OPC in mice in GF conditions (WT-GF, aged 6–9 weeks, both sexes). In contrast to WT-SPF mice, WT-GF mice exhibited substantial oral fungal loads of *C*. *albicans* at 5 days p.i., at levels similar to Abx-treated animals (~10^3^ CFU/g, Figure 2A). As a control for infection, mice lacking the ability to respond to IL-17 (*Act1^–/–^*, housed in SPF conditions) were confirmed to be susceptible to OPC, at levels somewhat higher than GF- or Abx-treated mice (~10^4^ CFU/g). The percentages of mice that cleared the infection corresponded to fungal loads; that is, more than 90% of GF mice had detectable fungal loads on day 5 p.i., whereas none of the SPF mice harbored detectable fungi in the tongue (Figure 2A). Thus, the presence of the microbiome is required for clearance of *C*. *albicans* from the oral mucosa in response to a first encounter.

Despite the complexity of the microbiome, a single obligate anaerobe, SFB, has been shown to be sufficient to drive accumulation of IL-17–expressing cells within the ileum and small intestinal lamina propria (49) and is a normal constituent of many animal facilities, including ours. Therefore, as expected, Abx treatment reduced SFB levels in fecal pellets from SPF mice (Figure 2B), correlating with OPC susceptibility. However, SFB is not detectable within the oral mucosa (28, 50) (Supplemental Figure 1A; supplemental material available online with this article; https://doi.org/10.1172/jci.insight.160348DS1). Accordingly, to determine if SFB that is selectively gut resident can influence immune events in the oral mucosa, WT-GF mice were given SFB (or PBS control) by gavage. Fourteen days later mice were infected with *C*. *albicans*, with oral fungal loads assessed on day 5. Most mice colonized with SFB cleared the infection, with average fungal loads approximately 100-fold less than GF mice receiving PBS (Figure 2A). Concomitantly, oral *Il17a*, *Il22*, and *Defb3* levels were increased in SFB-colonized mice (Figure 2, C and D).

**Figure 3:**
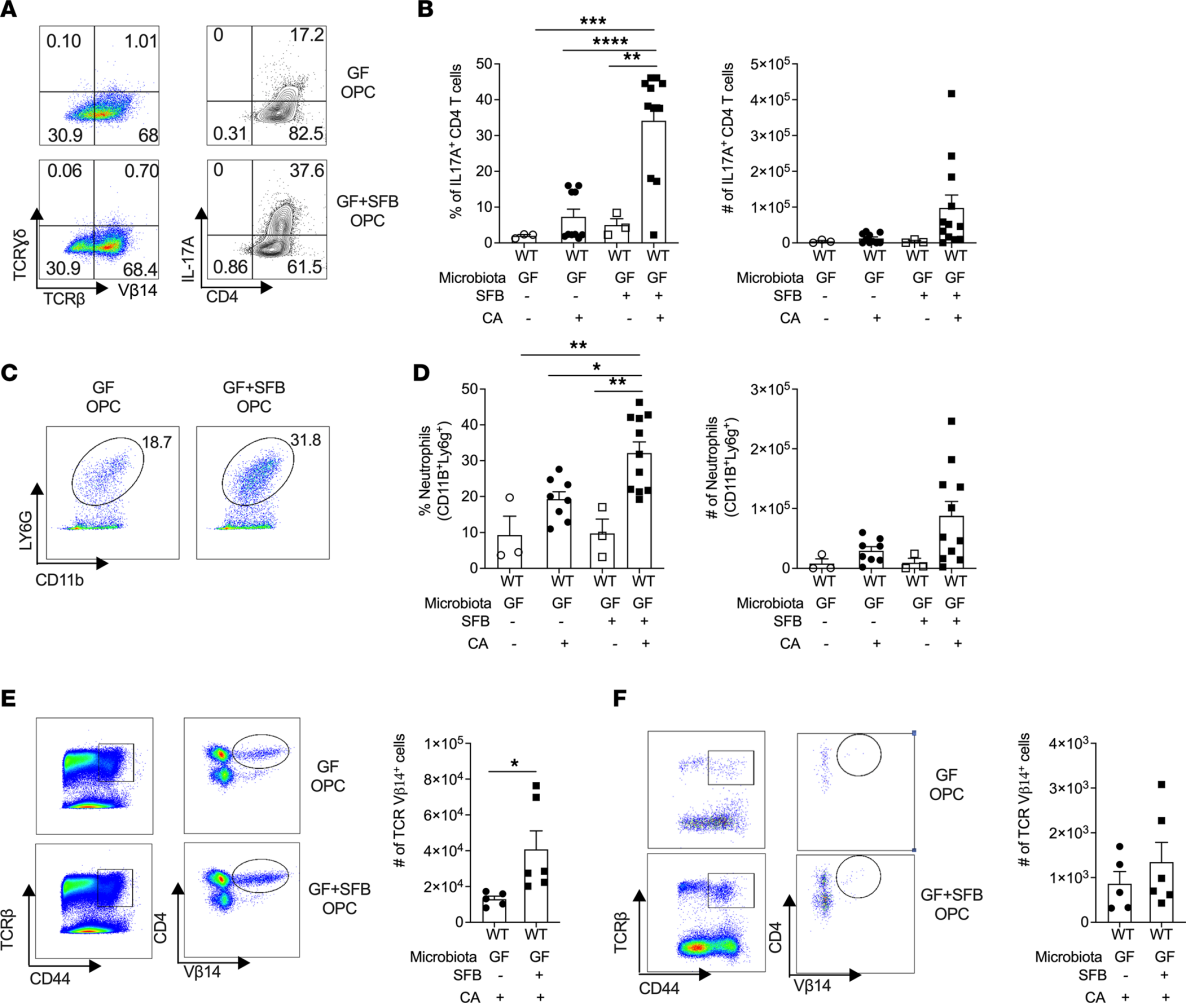
SFB mono-colonization in OPC triggers Th17 expansion and neutrophil recruitment. WT-GF mice were gavaged with PBS or SFB for 14 days followed by sublingual inoculation with *C*. *albicans*. Tongue homogenates were prepared at day 2 p.i., and (**A** and **B**) CD45^+^ cells were stained for TCRγδ, TCRβ, CD4, and intracellular IL-17A. (**A**) Representative FACS plot showing CD45^+^CD4^+^IL-17A^+^ cells. (**B**) Percentages and total numbers of CD4^+^IL-17A^+^ T cells. (**C**) Representative FACS plot showing CD45^+^CD11b^+^Ly6G^+^ cells. (**D**) Percentage and number of neutrophils. (**E** and **F**) CD45^+^ cells were stained for TCRβ, CD44, CD4, and Vβ14. *Left*: Representative FACS plot showing TCRβ^+^CD44^+^CD4^+^TCR Vβ14^+^ cells. *Right*: Total numbers of TCRβ^+^CD44^+^CD4^+^TCR Vβ14^+^ cells. Data from 1 experiment, *n* = 5–6. Graphs show mean ± SEM and data were analyzed by Student’s *t* test or ANOVA and Tukey’s multiple comparisons test. *P < 0.05, ** < 0.01, *** < 0.001, **** < 0.0001.

*C. albicans* is not a commensal microbe in mice, and hence the OPC model used here reflects the innate response to this fungus (44, 46, 51, 52). Upon this first encounter with *C*. *albicans*, IL-17 is expressed prominently in γδ T cells and a subpopulation of TCRαβ^+^ innate (“natural”) Th17 cells (53). These “natural” TCRβ^+^ cells are not *C*. *albicans* specific but undergo proliferative expansion in response to fungal hyphae (see details in Discussion) (44, 52–56). Indeed, there was a marked increase in the percentage and total number of IL-17^+^CD4^+^TCRβ^+^ cells in SFB–mono-colonized mice compared with GF animals (Figure 3, A and B). SFB did not alter total proportions of TCRβ^+^ or TCRγδ^+^ cells after *C*. *albicans* infection (Supplemental Figure 1, B and C), and frequencies of proliferating CD4^+^ T cells between groups were comparable (Supplemental Figure 1D). Thus, while SFB colonization did not alter the frequency of γδ^+^ T and TCRβ^+^ T cells in the tongue, SFB colonization increased the IL-17–producing ability of oral TCRβ^+^ T cells. Consistent with the induction of type 17 responses, neutrophil recruitment to the tongue in SFB-colonized mice was elevated compared with GF mice (Figure 3, C and D). SFB-specific Th17 cells uniformly express the Vβ14 TCR (57). As expected, there were increased numbers of TCR Vβ14^+^ cells in the mesenteric lymph nodes of SFB-colonized mice (Figure 3E). There was a similar trend of increased TCR Vβ14^+^ cells in the tongue of mice colonized with SFB and subjected to OPC, though the overall numbers of T cells in the oral mucosa were far lower than the intestine as previously described (53, 58) (Figure 3F). These results indicate that SFB drives expansion of type 17 cells in the oral mucosa, correlating with neutrophil recruitment and control of OPC.

### SFB mono-colonization drives multiple oral transcriptional programs.

**Figure 4:**
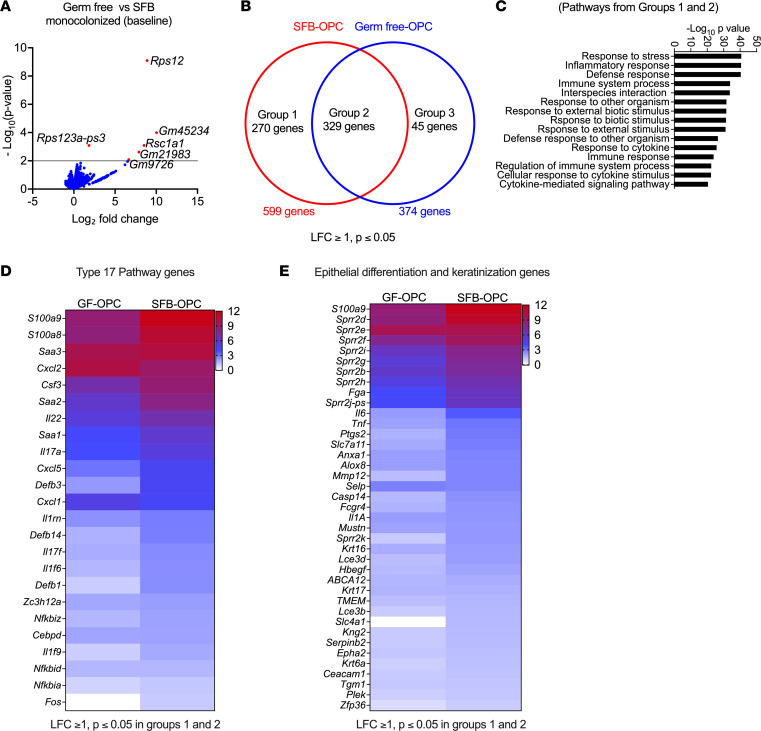
SFB mono-colonization modulates oral transcriptional responses during OPC. Whole tongue mRNA from GF and SFB–mono-colonized mice infected orally with *C*. *albicans* was harvested at day 2 p.i. and subjected to RNA-Seq analysis. (**A**) Volcano plot of transcriptional changes in sham (uninfected) GF versus sham (uninfected) SFB–mono-colonized mice at baseline. (**B**) Gene expression was normalized between GF-OPC and GF-Sham mice and SFB-OPC and SFB-Sham mice, respectively. Venn diagram of differentially regulated genes in *C*. *albicans*–infected SFB–mono-colonized versus GF mice. LFC, log fold change. (**C**) g:Profiler analysis of the top 15 GO biological processes inferred from differentially regulated genes in groups 1 and 2 (from **B**). (**D**) Heatmap of type 17 pathway genes in SFB-OPC versus GF-OPC groups normalized against their respective sham controls. (**E**) Heatmap of genes in epithelial repair and keratinization pathways in SFB-OPC versus GF-OPC groups normalized against their respective sham controls.

To gain a more comprehensive view of the impact of SFB on the oral mucosa, we performed transcriptome profiling of tongue from GF or SFB–mono-colonized mice (*n* = 4) at baseline (i.e., gavaged with PBS) or 2 days after oral *C*. *albicans* infection. Surprisingly few genes were differentially expressed in GF versus SFB–mono-colonized mice at baseline (Figure 4A), indicating that SFB exposure alone does not substantially alter overall transcriptional events in the oral mucosa. However, after oral *C*. *albicans* infection, a large number of genes were affected (Figure 4B), which we grouped into 3 categories: group 1 included transcripts induced only in SFB–mono-colonized mice during OPC; group 2 included genes induced during OPC in both SFB and GF mice; and group 3 included genes upregulated only in GF mice during OPC (Figure 4B). Gene Ontology (GO) pathway analysis of genes induced in mice colonized with SFB (i.e., groups 1 and 2) showed, as expected, that the overall response reflects inflammatory and immune responses or responses to stress (Figure 4C).

Notable among these gene sets were IL-17 signature genes such as antimicrobial peptides (*Defb1*, *Defb3*, *Defb14*, *S100a8/9*), neutrophil-recruiting genes (CXC chemokines, G-CSF/*Csf3*), and IL-17–related transcription factors or RNA binding proteins (NF-κB, AP-1, C/EBP family members, Regnase-1/*Zc3h12a*) (Figure 4D). Also included in the IL-17 signature gene list were acute-phase response genes encoding SAAs (*Saa1*, *Saa2*, *Saa3*), which also correlated with *C*. *albicans* clearance (Supplemental Figure 2, A–C). In this regard, SFB colonization is known to trigger SAAs, which in turn are potent inducers of Th17 cells (32, 59). Therefore, we were surprised that even mice lacking Saa1–4 (termed *Saa^–/–^*) were fully resistant to OPC (Supplemental Figure 2D), consistent with our previous observation that *Saa1/2* doubly deficient mice clear *C*. *albicans* from the mouth normally (15). Additionally, we also observed differential expression of gene sets involved in epithelial cell differentiation and tissue cornification (Figure 4E), which is in keeping with the major wound healing and tissue-reparative signals known to be highly active during OPC and regulated, at least in part, by IL-17 and IL-22 (15, 27, 60).

**Figure 5:**
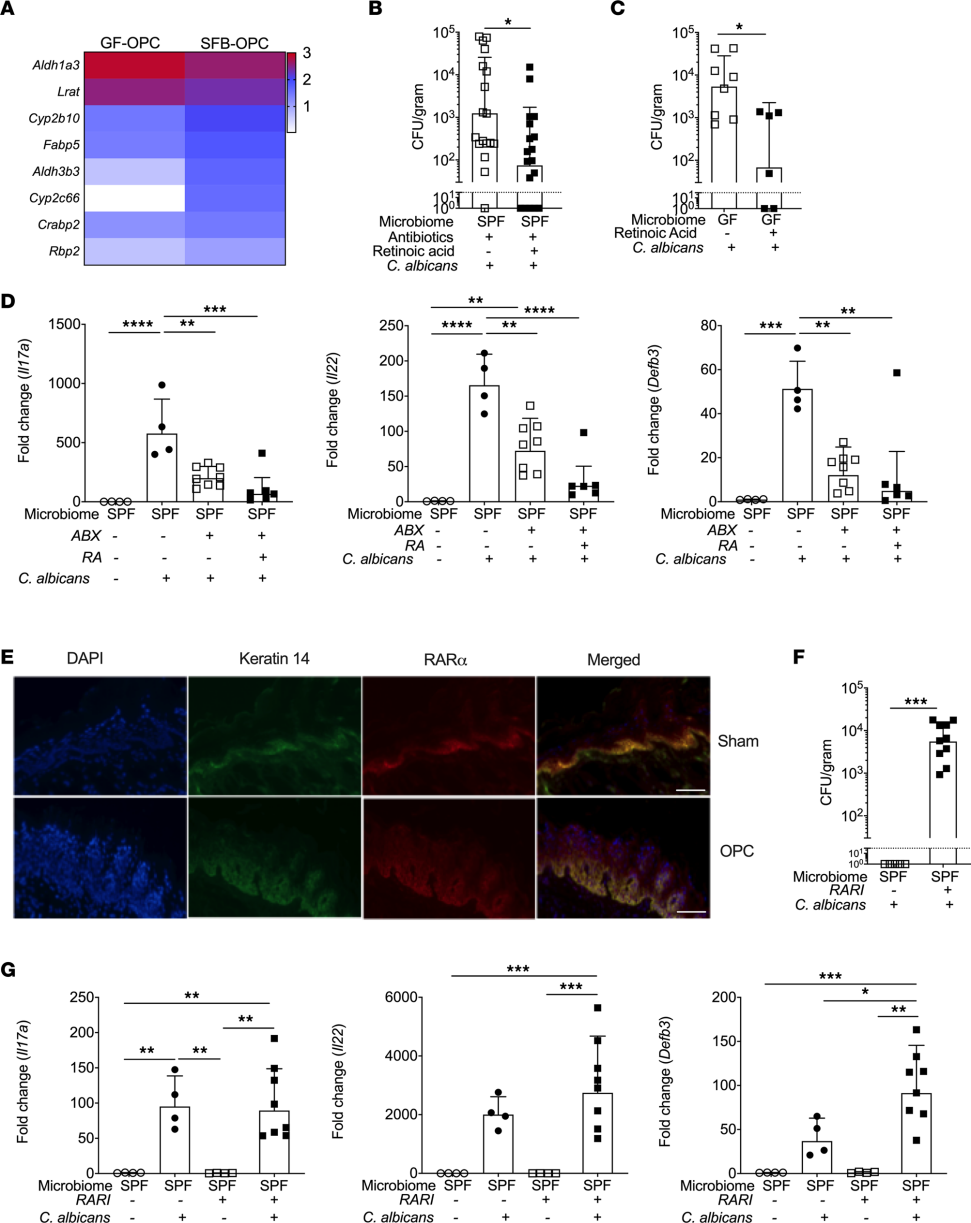
The RA pathway is induced by SFB mono-colonization and promotes immunity to OPC. (**A**) Heatmap showing selected genes in the RA/RAR pathway comparing GF-OPC and SFB-OPC mouse groups normalized to respective sham controls. (**B**) WT-SPF mice were given Abx for 7 days and infected orally with *C*. *albicans*. Mice were administered RA or vehicle i.p. starting day –6 relative to infection until day 5 p.i. Fungal burdens were assessed by plating and CFU enumeration. Data are from 2 independent experiments. Graphs show geometric mean ± SD analyzed by Mann-Whitney *U* test. (**C**) GF mice were administered RA or vehicle by oral gavage on alternating days starting day –6 until day 5 p.i. Fungal burdens were assessed by plating and CFU enumeration. Data are from 2 independent experiments. Graphs show geometric mean ± SD analyzed by Mann-Whitney *U* test. (**D**) WT-SPF mice were treated with Abx 7 days. Mice were administered RA or vehicle i.p. starting on day –6 until day 2 p.i. RNA from whole tongue at day 2 p.i. was analyzed by qPCR for indicated genes normalized to *Gapdh*. Graphs show mean ± SEM analyzed by ANOVA and Tukey’s multiple comparisons test. (**E**) WT-SPF mice were infected orally with *C*. *albicans* for 2 days. Tongue cryosections were stained for DAPI, K14, and RARα. Data representative of 3–5 mice per group. (**F**) WT-SPF mice were treated with RAR inhibitor (RARI; BMS493) or vehicle. Fungal burdens were assessed by plating and CFU enumeration. Graphs show geometric mean ± SD, analyzed by Mann-Whitney *U* test. Data pooled from 2 experiments. (**G**) WT-SPF mice were administered RARI or vehicle and infected orally with *C*. *albicans*. RNA from whole tongue at day 2 p.i. was subjected to qPCR for the indicated genes normalized to *Gapdh*. Graphs show mean ± SEM, analyzed by ANOVA and Tukey’s multiple comparisons test. Data pooled from 2 experiments. *P < 0.05, ** < 0.01, *** < 0.001, **** < 0.0001.

Another regulatory pathway involved in mucosal tissue repair is mediated by RA signaling through RAR transcription factors (39, 41, 61–63). Although retinoids have been shown to have direct antimicrobial or fungistatic activity on *Candida* species and to modulate macrophage responses (64, 65), comparatively little is known about the relationship between RA and OPC pathogenesis. In this regard, we also noted that a cluster of genes related to the RA pathway were altered in tongues of mice mono-colonized with SFB compared with GF mice (Figure 5A). These findings prompted us to ask whether RA administration could restore resistance to OPC in the context of microbiota insufficiency. Accordingly, we administered all-trans-RA to Abx-treated or GF mice prior to and throughout oral *C*. *albicans* infection. Indeed, RA led to substantially reduced oral fungal loads in both Abx-treated and GF mice (Figure 5, B and C). Since RA can drive Th17 cell development and function, we investigated the impact of RA on type 17 responses. Surprisingly, RA administration did not induce *Il17a*, *Il22*, or *Defb3* expression (Figure 5D). Thus, RA appears to confer additional antifungal activities in OPC in a manner independent of canonical type 17 responses.

To probe the role of RA/RAR signaling during OPC, we evaluated expression of RARα in oral epithelial tissue. It is known that during oral *C*. *albicans* infections, the superficial epithelial layer (SEL) of oral epithelium undergoes a process of sloughing that helps promotes fungal clearance, while the underlying basal epithelial layer (BEL, characterized by expression of keratin 14, K14) undergoes proliferation and differentiation programs that replace the SEL (66). Immunofluorescence imaging of tongues in uninfected (sham) or infected (OPC) mice indicated that RARα was expressed prominently in the K14^+^ BEL and to a lesser extent was visible throughout the suprabasal differentiating/postmitotic layers (Figure 5E). Expression of *Rara* mRNA was not altered on a per-cell basis according to RNA-Seq data (not shown), but immunofluorescence (IF) imaging revealed a clear expansion of the K14^+^ BEL that expresses RARα protein (Figure 5E). To determine whether RAR signaling mediates protection against OPC in a healthy host, WT-SPF mice were treated with the RAR inverse agonist (RARI; BMS493) (67). Consistent with the failure of RA treatment to restore type 17 cytokine expression (Figure 5, B and C), RAR inhibition markedly elevated oral fungal burdens (~10^4^ CFU/g) (Figure 5F) but did not impair induction of *Il17a*, *Il22*, or *Defb3* (Figure 5G). Thus, while RA/RAR activation mediates microbiome-dependent protection against OPC, its effects appear to be independent of type 17 activation.

## Discussion

Mice and humans lacking IL-17 receptor signaling pathway components are highly susceptible to OPC (9, 11). Antibiotic use is also associated with increased susceptibility to candidiasis, including both mucosal but also the more life-threatening systemic form of invasive *C*. *albicans* infection (25, 68). The last decade has seen an exciting upsurge of our understanding of the composition and influence of the microbiome on immune homeostasis, due in part to more widespread access to gnotobiotic facilities and advances in defining the composition of the bacterial microbiome.

Unlike humans, mice do not normally harbor *C*. *albicans* as a gut commensal microbe (69). However, long-term *C*. *albicans* gut colonization can be established using antibiotics or nonpathogenic strains, and non–*C. albicans* species are often found in laboratory mice (23, 70–73). Upon intestinal colonization, *C*. *albicans* has been shown to impact immune homeostasis, including generation of Th17 cells that can exacerbate lung and kidney inflammation but that can also mediate cross-reactive protection against invasive *C*. *albicans* and *Staphylococcus* *aureus* infections (23, 74, 75). Gut-associated *C*. *albicans* also orchestrates extraintestinal events, including a surprising observation of effects on promoting social behavior (76, 77). A recent report shows that long-term antibiotic use, specifically vancomycin that targets SFB, promotes susceptibility to invasive candidiasis in mice, which is linked to reduced Th17 cells and reduced diversity in the microbiome (68).

The discovery that a single component of the intestinal microbiome, SFB, was capable of eliciting local as well as long-range Th17 responses represented an advance in our understanding of how host immunity is shaped by commensal bacteria (49, 78). Since then, SFB has been shown to potentiate Th17 responses at multiple extraintestinal sites (36, 38, 40, 79–81). Moreover, SFB in the gut amplifies IL-17 production in a manner dependent on IL-17R signaling within the gut epithelium (37). In contrast, the influence of SFB on oral mucosal immunity is not well described, though a recent study demonstrated that SFB modulates alveolar bone homeostasis in a model of periodontal bone loss (50, 82).

The present study reveals a protective role for SFB residing in the gut to promote immunity to *C*. *albicans* in the mouth. In part this gut-oral axis is driven, as we expected, through upregulation of known IL-17–dependent antifungal pathways in the tongue, such as β-defensins and neutrophil-recruiting chemokines. Additionally, SFB-driven immunity appears to be mediated by RA, which is derived from vitamin A and carotenes and is a metabolite of SFB (39). In humans, vitamin A deficiency is linked to increased viral and bacterial infections, and vitamin A supplementation ameliorates disease (83). Our work thus reveals a type 17–independent link between RA and the intestinal microbiome in establishing protective antifungal immunity in the oral cavity.

Numerous studies have shown convincingly that SFB is not found in the mouth (28, 32, 50). Rather, intestine-resident SFB appears to establish a gut-oral axis that limits oral candidiasis. Commensal bacteria including SFB increase the availability of metabolites through de novo synthesis or breakdown of dietary products that can modulate local and distal immune responses (84, 85). Some of these metabolites, including short chain fatty acids and RA, can regulate development and function of Th17 cells. For example, ileal RA induced by SFB regulates Th17 cells and RAR signaling to promote *Citrobacter* *rodentium* clearance from the colon (39). Physiological concentrations of RA drive Th17 polarization (86, 87). In agreement with this, conditional loss of RARα in T cells abrogates Th17 differentiation, and consequently mice deprived of dietary vitamin A have fewer Th17 cells (88).

RA/RARα can amplify conventional (adaptive) Th17 cells, but its impact on innate T cells is not well understood. In the acute OPC model used here, innate-acting TCRαβ^+^ cells represent the primary source of type 17 cytokines (see below). These results indicate that in the context of acute (innately driven) OPC, both type 17 signaling and RA/RAR activation promote fungal clearance in parallel in an apparently independent manner. A similar observation was made in elegant studies of *C*. *rodentium*, where microbiome-dependent bacterial host defense was mediated by RA/RAR signaling but not type 17 responses (39).

SFB-dependent immunity against OPC did not correlate with changes in the proportions of CD4^+^ T cells or proliferating CD4^+^ T cells, and T cells in the oral mucosa are present at very low frequencies compared with the gut (53, 58). There was a trend of increased T cells bearing the Vβ14 TCR, which is linked to SFB recognition (57), but it is still unclear whether SFB-specific T cells generated in the gut traffic to the mouth. We have previously demonstrated that upon first encounter with oral *C*. *albicans*, IL-17 is expressed almost entirely by tongue-resident γδ^+^ T cells and a population of innately acting CD4^+^TCRβ^+^ cells, sometimes termed “natural Th17 cells” (nTh17 cells) (52, 53, 89). It has been suggested that type 3 innate lymphoid cells may play a role as well (90–92), though they are extremely rare in the mouth and *Rag1^–/–^* mice are highly sensitive to OPC (46, 53, 93). It should be noted that the oral nTh17 cells that were activated in the 2- to 5-day time frames assessed here are not *Candida* specific; they exhibit a varied TCR repertoire and do not activate TCR signaling, measured using a Nur77 reporter system that reports TCR signaling strength and duration (46, 52, 53, 93, 94). Moreover, nTh17 cells undergo a local proliferative expansion, which is independent of CCR6 or CCL20, suggesting that T cells are not recruited to the tissue (52). In this regard, SFB has been shown to induce SAA expression in the intestinal epithelium and promote expansion of adaptive Th17 cells (32, 49), and dietary vitamin A modulates SAA expression through RAR signals (95). Even though SAAs were decreased in tongues of mice subjected to microbiome depletion and increased upon SFB reconstitution, mice lacking SAA1–4 were resistant to OPC. One potential explanation is that SAAs promote inflammatory rather than protective Th17 cells (31, 59). SAAs also bind RA and promote transport and adaptive immunity (41), but our data suggest that SAA-mediated RA delivery is apparently not enough to mediate oral antifungal immunity. Adaptive, antigen-specific T cell responses can be generated in immunocompetent SPF mice, which are dominantly Th17 and provide an added layer of immune benefit on top of the innate immune response (44, 46). It will be interesting to determine the impact of the microbiome/SFB on this adaptive response.

Another unexpected observation was that SFB colonization in GF mice caused only negligible changes in homeostatic transcriptional responses (5 genes in total). Nonetheless, colonization with SFB provoked marked gene expression changes after only 2 days of exposure to fungus. Since SFB is not present in the oral mucosa (28, 82), this increased transcriptional landscape is not due to direct bacterial contact with the oral epithelium. Instead, we postulate that SFB in the gut primes distal responses to *C*. *albicans* in the oral epithelium. Indeed, SFB can induce altered chromatin states in RAR transcriptional targets that lead to *C*. *rodentium* clearance from the colon, even though SFB attaches to epithelia within the ileum (39). Moreover, it appears that the effects of RA/RAR signaling are not via the IL-17/IL-22 pathway, suggesting these events are combinatorial rather than interdependent.

The observed increases in genes associated with epithelial repair, differentiation, and keratinization in SFB–mono-colonized mice infected with *C*. *albicans* were consistent with the known capability of RA to promote epithelial barrier integrity (96). SFB-induced RA/RAR signaling likely drives oral epithelial differentiation and repair during OPC, commensurate with the high expression of RARα in the K14^+^ BEL. We showed that replenishment of *C*. *albicans*–infected superficial epithelial cells was an essential contributor to OPC host defense, mediated by IL-22/STAT3 signaling on the BEL (15). Additional genes in the epithelial repair gene sets encoded members of the small proline-rich 2 family (*Sprr2*). Interestingly, *Sprr2* proteins are implicated in epithelial differentiation downstream of RA/RAR signaling, and an antimicrobial role against Gram-positive bacteria has been described for *Sprr2a* in the intestine (97). It is tempting to speculate that *Sprr2* may contribute to *C*. *albicans* host defense by one or both of these mechanisms, but further studies are needed in this regard.

In addition to OPC, antibiotic use is associated with vulvovaginal candidiasis (VVC) (98). Unlike OPC, *C*. *albicans* infections in the vaginal tract result from excessive inflammation, and the role of IL-17/Th17 signaling in this disease setting is far less compelling (99–101). It will be very intriguing to see if SFB and/or RA can similarly rescue immunity to VVC.

Together, this work shows that a single component of the gut microbiome can serve as critical initiator of the oral immunosurveillance program against *C*. *albicans*. Candidiasis in the mouth as well as the vaginal tract are commonly observed with antibiotic use. This study suggests that RA as a metabolite might be a strategy to ameliorate or limit OPC in dysbiotic settings. Moreover, these findings raise possibilities for other commensal metabolites that might be harnessed to promote systemic immune responses in the face of emerging fungal resistance.

## Methods

### Study design.

This study interrogated the roles of microbiome components in oral antifungal immunity. We used a standard mouse model of oral *C*. *albicans* infection in gnotobiotic or SPF mice reconstituted with the bacterium SFB.

### Mice.

GF or SPF mice on the C567BL/6 background were from the University of Pittsburgh gnobiotic core or Taconic Farms, respectively. *Act1^–/–^* mice were from Ulrich Siebenlist (deceased, NIH, Bethesda, Maryland, USA), and *Il17ra*^–/–^ mice were a gift from Amgen Corporation (Thousand Oaks, California, USA), both available under a material transfer agreement. *Saa^–/–^* mice [C57BL/6-Del(7Saa3-Saa2)738Lvh/J] were from The Jackson Laboratory (Bar Harbor, Maine, USA). Experiments were performed on age-matched mice (6–9 weeks) of both sexes. Experiments were conducted in accordance with protocols approved by the University of Pittsburgh IACUC. Guidelines in the *Guide for the Care and Use of Laboratory Animals* of the NIH (National Academies Press, 2011) were followed.

### OPC model.

Mice were subjected to OPC by sublingual inoculation with *C*. *albicans* (CAF2-1) or PBS-saturated cotton balls for 75 minutes under anesthesia as described (42, 45). Tongues were homogenized in C-tubes with a gentleMACS homogenizer (Miltenyi Biotec). Serially diluted homogenates were plated on YPD/Amp agar for CFU assessment. For microbiome depletion, mice were given sterile water containing ampicillin (1 g/L), metronidazole (1 g/L), neomycin (1 g/L), and vancomycin (0.5 g/L) for 7 days prior to *C*. *albicans* infection. IL-17A (1 μg), IL-22 (1 μg), or PBS was injected i.p. 12 hours prior to and 24 hours after infection. RA was given i.p. (300 μg or DMSO) in 100 μL corn oil on alternating days starting 6 days before and during oral *C*. *albicans* infection. For RAR inhibition, 400 μg BMS493 (Tocris Bioscience) suspended in (1:10) DMSO/corn oil was injected i.p. on alternating days starting 6 days before and during oral *C*. *albicans* infection.

### SFB colonization and quantitation.

Fresh fecal pellets from mono-colonized mice (housed at UPMC Children’s Hospital of Pittsburgh) were homogenized through a 100 mm filter, pelleted at 3400 rpm for 10 minutes (Thermo Fisher Scientific TX-1000 centrifuge), and resuspended in PBS. Mice were administered 1/4 pellet or PBS by oral gavage 14 days prior to OPC induction. To assess SFB DNA content, DNA from ileal and cecal contents was isolated using a Fast DNA stool kit (Qiagen). Bacteria were collected from buffered whole tongue and DNA was extracted. SFB levels were assessed by qPCR using 16S-Eubacteria and SFB-specific primer pairs. Ct values above baseline were averaged and normalized using ΔΔCt.

### qPCR, RNA-Seq, and g:Profiler analyses.

Whole tongue was homogenized using a gentleMACS Dissociator (Miltenyi Biotec), and total tongue RNA was extracted using RNeasy kit (Qiagen). Primers were from Quantitect Primer Assays (Qiagen). For RNA-Seq, cDNA libraries were prepared from whole tongue RNA (*n* = 4 mice per group) harvested at day 2 p.i. Nextera XT kit sequencing was performed on the Illumina NextSeq 500 platform by the Health Sciences Sequencing Core at the University of Pittsburgh. RNA-Seq data analysis was performed with CLC Genomics Workbench v. 22 (Qiagen Digital Insights) as described in the CLC Genomics manual. Briefly, quality control analysis was performed on FASTQ RNA-Seq reads. Reads with Phred quality score > 20 were aligned to the mouse reference genome (mm10, GRCm38.75) using default parameters. Analysis of differentially expressed genes (DEGs) was performed with default parameters. DEGs were filtered for biological and statistical significance at log fold change ≥ 1 and *P* ≤ 0.05. To assess enriched biological pathways due to DEGs, g:Profiler analysis was conducted using default parameters. RNA-Seq raw data files are available at the NCBI Sequence Read Archive (BioProject ID PRJNA865258).

### Histology and IF.

Cryosections were stained per Cell Signaling Technology recommendations. Antibodies SAA3 (catalog ab231680) and RARα (catalog ab275745) were from Abcam; goat anti-rat cyanine 3 (clone A10522), goat anti-mouse Alexa Fluor 488 (catalog A32723), and goat anti-rabbit cyanine 3 (catalog A10520) and K14 (clone LL002) were from Invitrogen. Images were acquired from at least 2 sections per mouse (*n* = 3–5 mice per group) on an EVOS FL microscope (Life Technologies).

### Flow cytometry.

Flow cytometry of tongue single-cell suspensions was performed as described with modifications (53). Tongue homogenates were prepared with a gentleMACS homogenizer and digested with DNase I (1 mg/mL; Roche) and collagenase IV (0.7 mg/mL) in RPMI (both from Invitrogen) at 37°C. Single-cell suspensions were separated by Percoll gradients. To assess IL-17A, single-cell suspensions were stimulated with PMA (50 ng/mL; Sigma-Aldrich) and ionomycin (5 μg/mL; Sigma-Aldrich) in the presence of GolgiPlug (Brefeldin A; BD Biosciences). After 3.5–4 hours, cells were stained for surface markers, and cytokines were detected with an intracellular staining kit (BD Biosciences).

### Statistics.

Data were analyzed on Prism (GraphPad). Fungal load data are presented as geometric mean ± SD and analyzed by 1-way ANOVA post hoc Tukey’s test or Mann-Whitney *U* test. FACS and qPCR data were analyzed by 1-way ANOVA with multiple comparisons or 2-tailed Student’s *t* test. Each symbol represents data from 1 mouse unless otherwise noted. A *P* value less than 0.05 was considered significant. *P < 0.05, ** < 0.01, *** < 0.001, **** < 0.0001.

### Study approval.

All the experiments were conducted following NIH guidelines under protocols approved by the University of Pittsburgh IACUC (Protocol 20097969, PHS Assurance Number D16-00118).

## Author contributions

FEYA and SLG conceived the study; FEYA, CZ, DAA, JM, VMB, TWH, and SLG developed methodology; FEYA, MB, CZ, DAA, JM, TCT, and VMB investigated; FEYA and SLG wrote the manuscript; FEYA, TWH, and VMB edited the manuscript; and SLG and TWH supervised the study.

## Supplementary Material

Supplemental data

## Version 1 09/22/2022

Electronic publication
