# Supplementary material for: A gut-oral microbiome–driven axis controls oropharyngeal candidiasis through retinoic acid
Source: JCI Insight. 2022 Sep 22;7(18):e160348. doi: 10.1172/jci.insight.160348 (PMC9675558; doi:10.1172/jci.insight.160348)
Supplement: Supplemental data [file jciinsight-7-160348-s105.pdf]

SUPPLEMENTARY INFORMATION

A gut-oral microbiome-driven axis promotes mucosal immunity to *Candida albicans* through retinoic acid

Felix EY Aggor [1], Martinna Bertolini [1,2], Chunsheng Zhou [1], Tiffany C. Taylor[1], Darryl A. Abbott, Javonn Musgrove [3], Vincent M. Bruno [4], Timothy W. Hand [3], Sarah L. Gaffen [1¶]

[1] Division of Rheumatology & Clinical Immunology, University of Pittsburgh, Pittsburgh, PA

[2] Department of Periodontics, School of Dental Medicine, University of Pittsburgh, Pittsburgh, PA

[3] RK Mellon Institute for Pediatric Research, Pediatrics Department, Infectious Disease Section, UPMC Children's Hospital of Pittsburgh, University of Pittsburgh, Pittsburgh, PA

[4] Institute for Genome Sciences, University of Maryland School of Medicine, Baltimore, MD

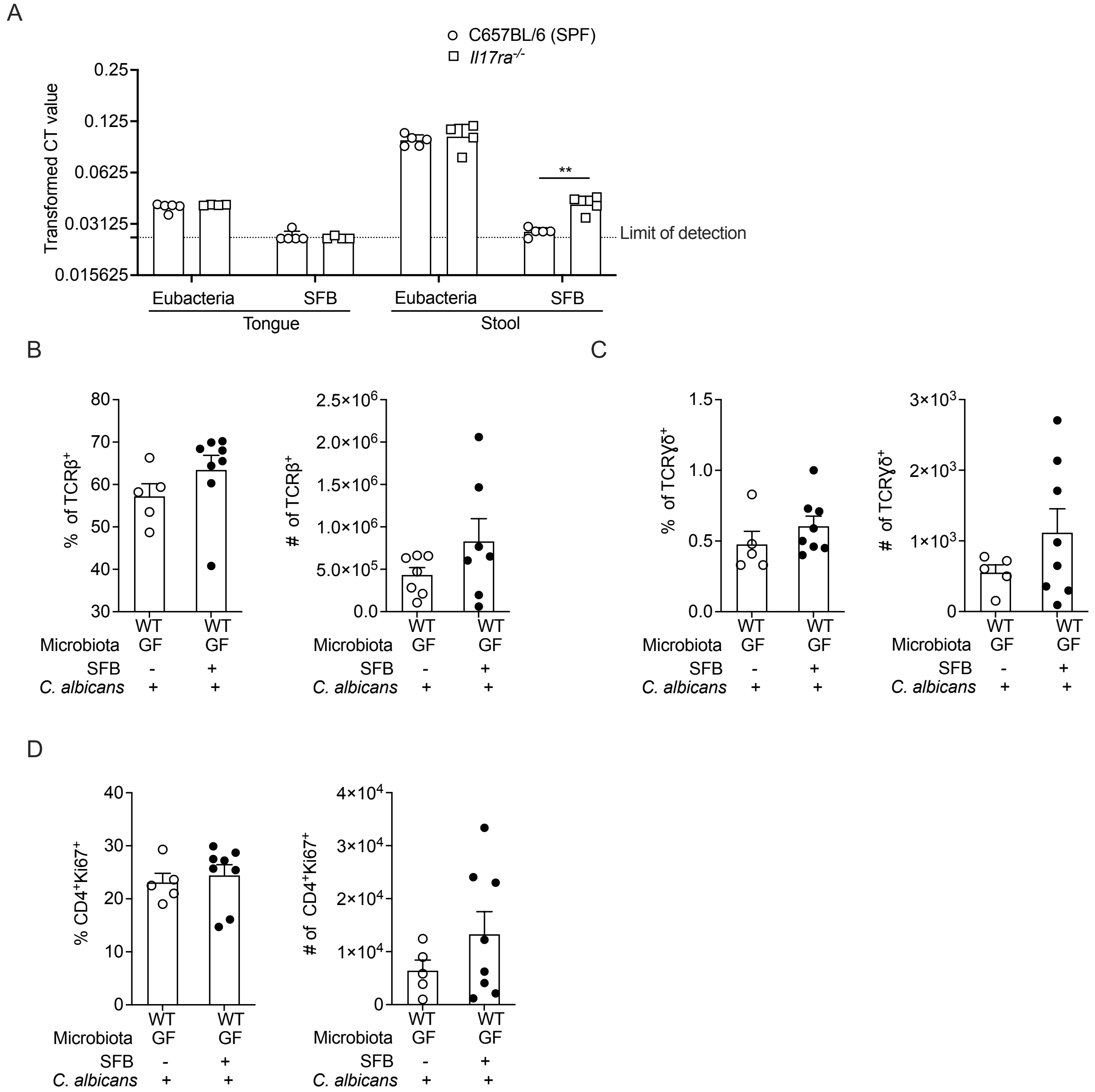

Supplementary fig. 1: SFB is not detected in murine tongue

(A) Tongue and stool samples from WT C56BL/6 mice (JAX) or *Il17ra*$^{-/-}$ mice were evaluated for SFB or total Eubacteria by qPCR. Dashed line = limit of detection (LOD). Percentage and numbers of tongue TCRβ^{+} (C) and γδ^{+}T cells (D) from *C. albicans*-infected GF or SFB mice harvested day 2 p.i. (E) Percentage and numbers of proliferating Tongue T cells (CD4^{+}Ki67^{+}) from *C. albicans*-infected GF and SFB mice harvested day 2 p.i.

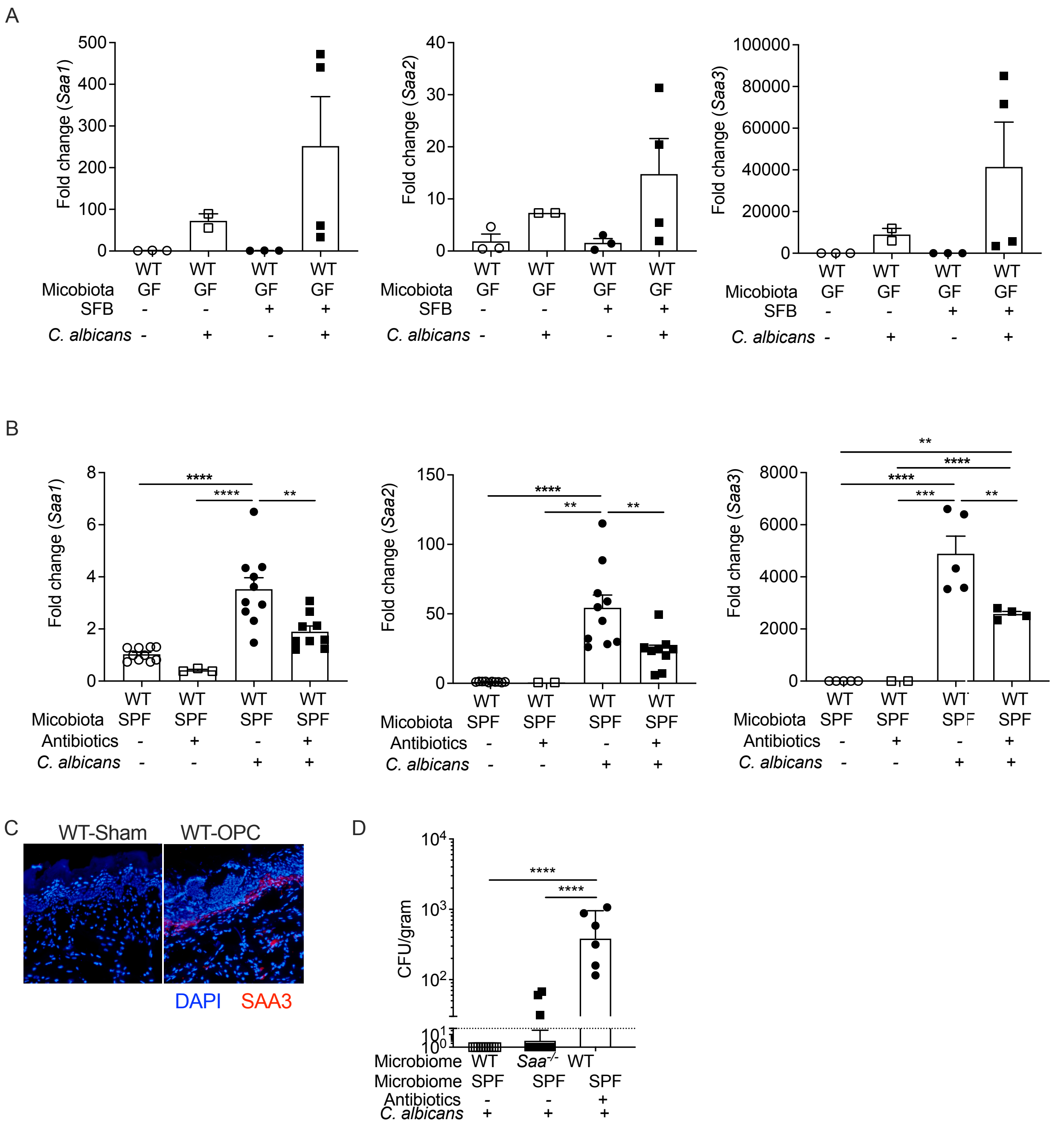

Supplementary fig. 2: Serum amyloids are induced during OPC but are not required for fungal clearance.

(A) WT-GF mice were gavaged with PBS or SFB, and after 14 days infected orally with *C. albicans*. Tongue homogenates prepared on day 2 p.i. were evaluated for the indicated mRNAs by qPCR, normalized to *Gapdh*. (B) WT-SPF mice were given antibiotics in drinking water for 7 days and then infected orally with *C albicans*. Tongue homogenates prepared on day 2 p.i. were evaluated for the indicated mRNAs by qPCR, normalized to *Gapdh*. (C) Frozen tongue sections from Sham (PBS)-infected or *C. albicans*-infected mice day 2 p.i. were stained for DAPI and

SAA3. (D) The indicated mice were given antibiotics in drinking water for 7 days and then infected orally with *C albicans*. After 5 d, fungal loads in tongue were evaluated by CFU enumeration of tissue homogenates. Graphs show geometric mean ± SD analyzed by ANOVA with Mann Whitney U. Data from 2 independent experiments.
